# Surgical treatment of bony nasal airway stenosis in a patient with adult Crouzon’s syndrome

**DOI:** 10.1093/jscr/rjac358

**Published:** 2022-07-30

**Authors:** Tatsuhiko Kamikonya, Go Inokuchi, Shun Tatehara, Mitsuko Yui, Ken-ichi Nibu

**Affiliations:** Department of Otolaryngology-Head and Neck Surgery, Kobe University Graduate School of Medicine, Kobe, Japan; Department of Otolaryngology-Head and Neck Surgery, Kobe University Graduate School of Medicine, Kobe, Japan; Department of Otolaryngology-Head and Neck Surgery, Kobe University Graduate School of Medicine, Kobe, Japan; Department of Otolaryngology-Head and Neck Surgery, Kobe University Graduate School of Medicine, Kobe, Japan; Department of Otolaryngology-Head and Neck Surgery, Kobe University Graduate School of Medicine, Kobe, Japan

**Keywords:** nasal airway stenosis, choanal atresia, craniosynostosis, Crouzon’s syndrome, sleep apnea

## Abstract

Crouzon’s syndrome is associated with the respiratory impairment of the upper airway due to mid-facial hypoplasia. We managed an adult Crouzon patient who wanted us to treat his choanal and nasopharyngeal stenosis for obstructive sleep apnea relief and tracheostomy tube extubation. We drilled out the abnormal maxillary bone and created a new nasal passage to the pharynx. Epithelialization of the new nasal cavity was completed within a month, and the patient was able to breathe through the nose and his sense of smell improved somewhat after the surgery. Although the apnea-hypopnea index had decreased, sleep apnea remained.

## INTRODUCTION

Crouzon’s syndrome is a congenital disorder characterized by craniosynostosis, which leads to premature fusion of the calvarian coronal sutures, resulting in skull and facial deformities. Crouzon’s syndrome is associated with intracranial pressure elevation, skull deformity and upper airway stenosis due to midface hypoplasia, although it is well known that there are phenotypic differences between individuals [1].

Midface hypoplasia is a feature of syndromic craniosynostosis, which is seen in syndromes such as Apert, Crouzon and Pfeiffer syndrome. Approximately, 68% of the patients with syndromic craniosynostosis reportedly develop obstructive sleep apnea (OSAS). The obstructive apnea-hypopnea index (AHI) of such patients often decreases as they grow up but not in patients with midface hypoplasia [2].

Treatment of Crouzon’s syndrome is challenging because few previous relevant reports can be found in the literature and due to the great variety of disease phenotypes. While many surgical approaches for treatment have been attempted, such as midface advancement using Le Fort III or Le Fort II plus zygomatic repositioning, during the early growth stages, there are no reports about the use of these surgical procedures for adult patients. Following a report on successful endoscopic surgery for congenital bony nasal stenosis in Aper and Peiffer syndromes [3], we decided to drill out the abnormal maxillary bone via gingival incision to provide relief for OSAS resulting from the Crouzon’s syndrome.

**Figure 1:**
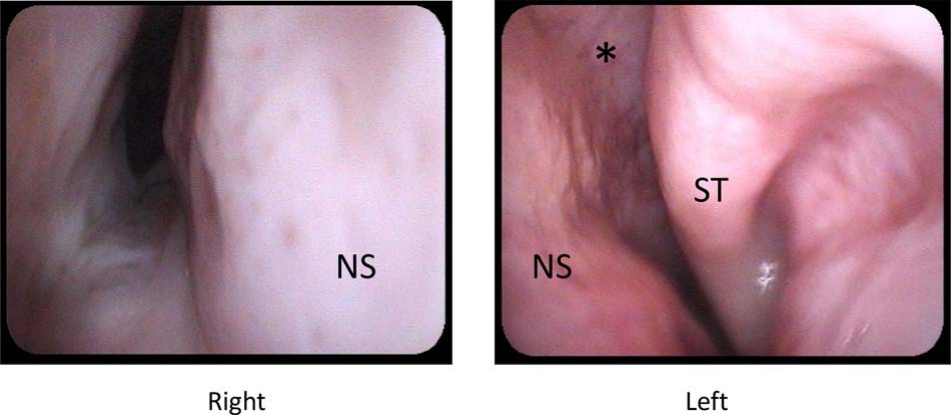
Preoperative nasal endoscopy; right nasal cavity is narrowed due to deviated nasal septum and fused maxillary and palatal bone; middle and inferior turbinates were deficient; superior turbinate and olfactory cleft were noted; abbreviations: ST, superior turbinate; NS, nasal septum; ^*^, olfactory cleft.

## CASE REPORT

**Figure 2:**
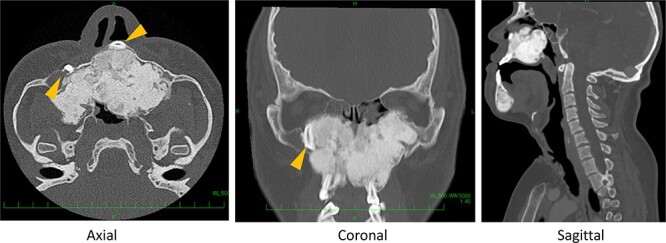
Preoperative computed tomography (CT) shows severe bone formation with inversed teeth (arrow heads); space between nose and pharynx was narrow.

**Figure 3:**
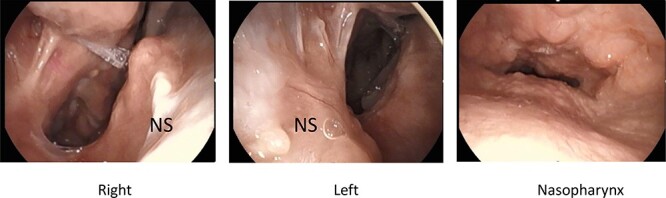
Post-operative nasal endoscopy; new nasal cavity has been created; posterior nasal septum and bilateral inferior and middle turbinates were defective; nasopharyngeal closure was incomplete because of submucosal cleft palate, but no exacerbation of regurgitation was observed after the surgery.

**Figure 4:**
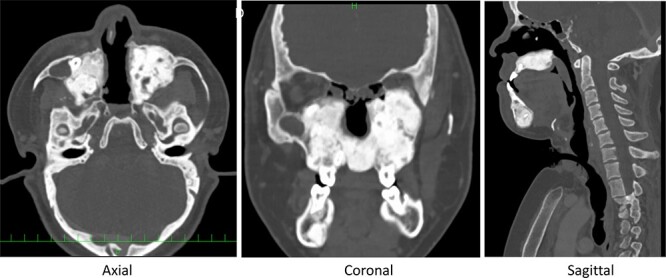
Post-operative CT midline bone has been drilled out, and space for nasal airflow has been widened.

A 25-year-old male with Crouzon’s syndrome was referred from our pediatric surgery department for the treatment of nasal cavity stenosis. The patient had a history of a previous tracheostomy at birth and three surgeries for nasal cavity stenosis in his childhood. After he had reached adulthood, he developed tracheal stenosis resulting from long-term tracheal intubation and nasal cavity stenosis. At his first visit, subglottic tracheal stenosis and nearly total blockade of the nasal due to cavity stenosis caused by abnormal maxillary and palatal bone were observed (Figs 1 and 2). Polysomnography following trial closure of the tracheal tube revealed moderate OSAS with an AHI of 18.8. Inspiratory and intravenous olfactometry showed no response and the patient had never experienced smelling anything. We drilled out the fused maxillary and palatal bone via the gingival approach under general anesthesia. As the posterior nasal cavity was completely blocked by the abnormal fused bone, we removed the bone until the pharynx could be reached. Exposed bone was covered with polyglycolic acid sheet and fibrin glue. The post-operative course was uneventful and the exposed bone had epithelialized within 1 month. The patient could breathe through his nose and smell something post-operatively, but he could not identify what he was smelling. Although polysomnography after 4 months showed a reduction in AHI (14.5), the tracheal stenosis was still too severe for the removal of the tracheal tube. No restenosis has occurred for 2 years after the surgery without any stenting (Figs 3 and 4).

## DISCUSSION

Crouzon’s syndrome is inherited in an autosomal dominant manner, but the extent of upper airway stenosis varies between individuals because of the phenotypic variance of Crouzon’s syndrome. While some reports of OSAS in Crouzon’s syndrome in infancy have been published [4, 5], no adult cases have been reported as far as could be ascertained.

Schafer identified maxillary hypoplasia and narrow posterior choanal openings as the most important factors in the pathogenesis of the upper airway obstruction of children with craniofacial anomalies [6]. A recent study of airway growth in Crouzon’s syndrome revealed that the reduction in the nasal airway volume would become nearly normal after 6 years of age but that the reduction of the pharyngeal airway volume would remain throughout adulthood [7]. The degree of improvements in OSAS by creating the nasal cavity alone in this patient with both mid-facial hypoplasia and choanal atresia was unpredictable. Eventually, AHI was reduced from 18.8 to 14.5, but the reduction was minor. Considering the comparatively less invasive nature of this technique and the continued use of positive airway pressure, this surgery to create nasal airflow seems to be adequate. Although stenting in choanal surgery during infancy is common [8], there was no restenosis in our case without stenting, probably because the choanal space we created was sufficient.

Although olfaction in Crouzon’s syndrome is rarely evaluated, anosmia of a 15-year-old woman and a 11-year-old male with Crouzon’s syndrome was reported to have improved to some extent after cranio-facial reconstruction [9]. As for the critical period for language development [10], nasal airflow might be essential for the development of a sense of smell in childhood. After the surgery, our patient could breathe through the nose and smell something, but eventually, he could not identify what he was smelling. Earlier surgery in childhood may be advisable for improved olfaction.

## CONCLUSION

Creating the nasal airflow in adults with Crouzon’s syndrome by drilling out the abnormal bone via the gingival approach proved to be a safe, simple and less invasive procedure. No restenosis was observed even though no stenting had been used. However, this surgery had only a limited effect on OSAS relief because of the narrow pharyngeal airway. Olfactory identification remained problematic even after the patient could breathe through his nose.
